# Growing inequality during the Great Recession: Labour market institutions and the education gap in unemployment across Europe and in the United States

**DOI:** 10.1177/00016993221083226

**Published:** 2022-03-28

**Authors:** Caroline Berghammer, Alicia Adserà

**Affiliations:** Department of Sociology, 27258 University of Vienna, Rooseveltplatz 2, 1090 Vienna, Austria; Vienna Institute of Demography (OeAW), Wittgenstein Centre for Demography and Global Human Capital (IIASA, OeAW, 27258 University of Vienna), Vienna, Austria; Princeton School of Public and International Affairs, 6740 Princeton University

**Keywords:** unemployment, education, labour market institutions, inequality, temporary employment, public sector employment, EPL

## Abstract

We study how the education gap in unemployment has evolved by gender and age groups across 28 European countries and the United States from 2000 to 2014, using the European Union's Labour Force Surveys and the US Current Population Surveys. During and after the Great Recession, the absolute education gap in unemployment expanded in almost all countries, which was mainly driven by a marked increase in the unemployment risk among low educated men. A two-step multilevel analysis confirmed the negative relationship between the education gap and both (lagged) GDP growth and GDP level. Further, institutional labour market features moderated the impact of the business cycle. A higher share of temporary employment boosted employment for less educated persons, thus flattening the education gradient in unemployment, while a larger public sector somewhat protected more highly educated individuals against unemployment. The gap for young workers was large in settings with strict regular contract regulations.

## Introduction

Recessions have detrimental socio-economic consequences, including increased social inequality that arises from their stronger effects on lower-educated individuals and families compared to their higher-educated counterparts. Empirical evidence on the 2008 Great Recession indeed shows that job loss was concentrated at the lower end of the educational distribution in both Europe (OECD, 2010) and the United States (Hoynes et al., 2012). One reason is the higher prevalence of low-educated persons in sectors that are most volatile across the business cycle (e.g. manufacturing or construction) and their overrepresentation in the temporary workforce. This growth in economic inequality is relevant considering its social consequences, which may ultimately erode social cohesion in a society (Van de Werfhorst and Salverda, 2012). Unemployment has been prominently linked to a number of adverse outcomes including lower life satisfaction, health and political participation (Gallie et al., 2001; McKee-Ryan et al., 2005; Paul and Moser, 2009). Those outcomes, in turn, create a greater distance between groups (beyond their economic standing), and also contribute to reproducing inequality in the next generation. A growing educational divide could also speed up the long-term trend of decline in occupations held by medium- and low-educated workers, and the parallel growth of occupations performed by the highly educated, leading to growing job polarisation (OECD, 2013c; Autor, 2014; Goos et al., 2014; Smeeding et al., 2011). Indeed, research from the US notes that the Great Recession accelerated this long-term trend (Smeeding et al., 2011).

While prior research has shown that the education gap in unemployment increased during the Great Recession (OECD, 2010; Grusky et al., 2011), which ended a period of stability across Western Europe from the 1990s until 2007 (Gebel and Giesecke, 2011), this paper first extends previous research to include the post-recession period. Second, it conducts a two-step multilevel analysis to explore what moderates the impact of the economic cycle on the education gap in unemployment. The aim is to provide a more nuanced picture of the factors that influence changes in the education gap in unemployment. This aligns with previous comparative work across OECD countries that shows the importance of how institutional labour market features interact with macro shocks (Gebel and Giesecke, 2011; Bertola et al., 2007; DiPrete et al., 2006). This paper includes 28 European countries and the United States (US), spanning from 2000 to 2014 (the first year where unemployment fell across the European Union after the recession). The first stage uses logistic regression models to estimate the unemployment gap across individuals according to educational attainment in each country and year for the whole sample and, separately, by gender and age group. These estimated gaps are subsequently used as the dependent variable in second-stage macro-level models for the panel of all 29 countries. GDP changes and GDP level are both used as indicators of the strength of economic fluctuations. In terms of institutional characteristics, the analysis considers the prevalence of temporary and public employment as well as the stringency of employment protection legislation.

The paper studies how the education gap evolved separately for men and women and in different age groups. First, the Great Recession has been dubbed a “male recession,” as many typically male-dominated jobs such as those in construction and manufacturing disappeared (Arpaia and Curci, 2010), which affected the education gap in unemployment differently among men and women. From a social relevance perspective, it is important to focus on gender because of how the socio-economic consequences of a man's job loss may differ from those of a woman. Given that men are often the main breadwinners, families tend to be more adversely affected by income drops ensuing from male job loss than female job loss, despite these consequences being most acute when both partners are unemployed (Grotti and Scherer, 2014). In addition, the negative consequences of men's unemployment on relationship quality are often more pronounced than women's unemployment because of how the breadwinner role is central to masculinity (e.g. Melzer, 2002). Second, analysing different age groups in this study was prompted after observing that young people were especially hard-hit by the Great Recession (e.g. Bell and Blanchflower, 2011a). Young people have faced significant barriers to job market entry (including those with college degrees) and gaining stable jobs. The socio-economic consequences of youth unemployment concern family decision-making, since it tends to postpone leaving the parental home, marriage and entry into cohabitation (Sobotka et al., 2011) and (further) delay or forego having children (Kreyenfeld and Andersson, 2014; Sobotka et al., 2011; Adsera, 2011). Middle-aged and older individuals, by contrast, may face severe downward mobility upon becoming unemployed. At the same time, they are more likely to have large financial commitments (e.g. housing, loans) and tend to be less flexible and mobile than their younger counterparts when searching for a new job. Older individuals (i.e. those nearing retirement age), in particular, might drop out of the labour force if it is too challenging to find a new job (Van Horn et al., 2011). Further, middle-aged and older individuals are more likely than their younger peers to have formed their own family, which means that crossover effects of unemployment to the spouse and to children are likely (Kalil, 2013; Brooks-Gunn et al., 2013; Schneider et al., 2016).

The paper is structured as follows: First, it outlines reasons for why recessions tend to increase the unemployment gap between educational groups and states the research hypotheses. The next section introduces the data and methods. This is followed by a descriptive section that shows trends in unemployment across educational attainment and a discussion about the estimates from the macro-level models. The concluding section summarises the main results.

## Educational inequalities across the business cycle and labour market settings

While low-educated people generally have weaker labour market prospects than those with more education, recessions are bound to widen this gap. This disproportionate effect on those with lower education during economic downturns may be explained by a set of mechanisms: First, the least educated are usually overrepresented in sectors that are most volatile over the business cycle. During the Great Recession, in particular, a larger share of jobs were lost in manufacturing, construction, mining and quarrying than in other sectors (OECD, 2010). Second, low-skilled workers are often the first to be dismissed at the onset of any recession. Empirical evidence shows higher outflow rates of low educated workers compared to the highly educated in some contexts, e.g., the Netherlands (Gautier et al., 2002; Wolbers, 2000), but not in others, e.g., Germany (Pollmann-Schult, 2005). Highly educated workers are likely harder to replace because of both their strong firm-specific skills—related to a higher prevalence of on-the-job training (OECD, 2014)—and broad general skills. As a result, firms may temporarily downgrade employee status through working-time and pay reductions rather than dismiss them (Saunders, 1992; van Ours and Ridder, 1995). The risk of job loss also sees relatively greater increases among low-educated employees during economic downturns because they are more likely to hold (short-term) temporary contracts that are not renewed upon expiration by firms under strain. Third, individuals with high human capital have better re-employment chances in the job market than other job seekers; for example, studies find higher inflow rates for highly educated workers than for low-educated ones (OECD, 2013b). Differences in the incentive structure concerning unemployment benefit replacement rates (Nickell and Bell, 1995) and the efficacy of search strategies (for Austria, see Weber and Mahringer, 2008) across educational levels may also explain this gap. Moreover, during job-shortages, highly educated workers may decide to compete for lower skilled positions than they would during economic booms. This means the highly educated are likely to crowd out the low educated, as previously shown in Germany (Pollmann-Schult, 2005), the Netherlands (Gesthuizen and Wolbers, 2010) and Spain (Dolado et al., 2000). In such cases, entry only improves for the less-educated once economic recovery speeds up alongside fast GDP growth.

Based on these three factors, when comparing active individuals in each group, *the education gap in unemployment is expected to widen more during recessions among men than among women (hypothesis 1a)*: Men are employed more frequently in sectors that are heavily affected by the business cycle, while a higher share of women work in relatively sheltered areas like the public sector (e.g. as teachers, social workers, nurses) or may drop out of the market rather than become unemployed. Indeed, empirical evidence from the Great Recession shows that in Europe, women's business-cycle volatility would strongly increase if they were represented proportionally to men across different industries and sectors (OECD, 2009: 47). Similarly, the US sectoral composition of unemployment played a large role in the gender gap in unemployment (Sahin et al., 2010). *Across age groups, the education gap is expected to fluctuate with the business cycle more sharply among young workers than among middle- or old-aged ones (hypothesis 1b)*. Youth employment generally has greater cyclical sensitivity (Bertola et al., 2007; Bell and Blanchflower, 2011a). Young, low-educated persons face more barriers to labour market entry and are at a higher risk of becoming unemployed than their older, low-educated peers who may have accumulated greater labour market experience and hold better-protected and more stable jobs.

Across countries, institutional labour market settings may either subdue or amplify the impact of the economic cycle (DiPrete et al., 2006). Following labour reforms in the 1990s, temporary work started to expand in OECD countries and has continued to do so in recent years. This rise was especially pronounced in Southern Europe (Dolado et al., 2000). Temporary employment was more common in 2000 among lower-educated persons (13% across all countries studied here) than among medium- (7%) or higher-educated persons (10%). By 2014, this gap had grown slightly (low: 16%, medium: 11%, high: 11%). However, these differences are moderate and some countries have similar temporary employment rates between education groups or even higher rates among highly educated workers. As a general observation, the proportion of temporary workers drops in the first stage of a recession (due to higher outflow rates) and rises again, just after the trough (Holmlund and Storrie, 2002). Hence, as the economy slowly picks up, temporary positions may be the first opportunities for low-educated persons to re-enter the labour market (Holmlund and Storrie, 2002; Barbieri and Scherer, 2009). Nevertheless, temporary employment is not a desirable form of employment for many. Notably, it has been linked to a number of adverse outcomes, such as higher employment insecurity and lower job satisfaction (OECD, 2002), a higher risk of in-work poverty (Van Lancker, 2012) and lower career stability (Blanchard and Landier, 2002). A high share of temporary workers accept this arrangement because they are unable to find a permanent job (Bell and Blanchflower, 2011b). *Because temporary employment is more prevalent among lower educated persons, the education gap in unemployment is expected to be lower when the share of temporary employment is higher (hypothesis 2)*.

Beside the availability of temporary employment, the scope of the public sector may also affect the size of the education gap in unemployment along the economic cycle. Public sector jobs are generally permanent and protected, although some countries’ public sectors have recently seen a moderate increase in the share of temporary contracts, which have accompanied downsizing. In the context of the Great Recession, public sector jobs in areas like teaching, health and public administration were hit less and somewhat later in the period than private sector positions (for the US, see Folbre, 2011). In all countries and across all years examined in this paper, public sector employment (defined as public administration, education, health and social work) is much more common among women (37% of those employed) than among men (around 15%) and among highly educated persons (30%) than among medium (17%) and low-educated persons (10%). Most public employees in this paper's country panel are highly educated (57% across all 29 countries), while only a small minority are low educated (5%). As educational attainment increased in these countries over the observation period, the public sector shifted towards employing the highly educated. Therefore, highly educated employees in countries with large public sectors may have been better shielded from unemployment by their overall job stability than both their low-educated peers in the same country and the highly educated in countries with smaller public sectors and, hence, less access to such jobs. *Thus, the education gap in unemployment may be larger (even during and since the recession) in countries where public sector jobs are abundant (hypothesis 3)*.

An additional indicator of labour market regulation considers the strictness of employment protection legislation (EPL) for regular contracts. Similar to the policy changes in the late 1990s (Dolado et al., 2002; Blanchard and Landier, 2002), reforms to permanent employment legislation in OECD countries led to less stringent protection against dismissal for permanent workers after the start of the Great Recession. This occurred through cuts to severance pay or prolonged probationary periods for new hires (European Commission, 2015). A strict regular contract EPL may imply a greater reluctance among employers to make permanent hires, especially in times of crisis, which could be associated with a steeper educational gradient, as found in Gebel and Giesecke (2011). Strict permanent employment regulation may constitute a starker obstacle for (particularly the least-educated) younger workers (and women) attempting to enter the market and start a career than for older workers, who are shielded as insiders in their permanent jobs (Bertola et al., 2007). However, deregulation may ease those difficulties and narrow the unemployment gap. *The education gaps in unemployment are expected to be larger for countries with stricter employment protection legislation (hypothesis 4)*.

## Data and methods

This paper uses the European Union's Labour Force Surveys (LFS) and the Current Population Surveys (CPS) from the US for the period 2000–2014. The sample includes Western European countries (Austria, Belgium, France, Germany, Luxembourg, Netherlands, Switzerland), English-speaking countries (Ireland, United Kingdom, the US), Nordic countries (Finland, Iceland, Norway, Sweden), Southern European countries (Cyprus, Greece, Italy, Portugal, Spain), Central and Eastern European countries (Bulgaria, Czechia, Hungary, Poland, Romania, Slovakia, Slovenia) and Baltic countries (Estonia, Latvia, Lithuania). Besides good comparability across countries and over time, another advantage of the LFS is its high response rate (see table 1). In 11 out of 29 countries, respondents are required by law to participate in the surveys. Moreover, the large sample sizes ensure the reliable analysis of population subgroups, which is especially pertinent when studying conditions that are experienced by relatively small shares of the population like unemployment. The CPS is a comparable data source for the US, conducted by the Census Bureau for the Bureau of Labor Statistics. It is regularly used by the OECD alongside the LFS to compute labour force statistics; this study used the March files from the annual CPS.

**Table 1.** Overview of country samples.

Country	Size of analytic samples, 2000–2014	Participation compulsory (yes/no)	Non-response rate in % (mean of 2004–2014)^1,2^	Sampling rate in % (mean of 2004–2014)^2^
	(1)	(2)	(3)	(4)
Austria	924,823	Y	9	0.61
Belgium	503,398	Y	27	0.31
Bulgaria	355,314	N	19	0.63
Cyprus	199,153	Y	3	1.41
Czechia	789,436	N	20	0.60
Estonia	100,221	N	33	0.65
Finland	328,028	N	22	0.90
France	1,935,617	Y	18	0.22
Germany	1,546,217	Y	3	0.28
Greece	1,278,065	Y	16	0.83
Hungary	1,298,764	N	16	0.91
Iceland	77,279	N	18	1.90
Ireland	904,121	N	17	2.81
Italy	2,968,222	Y	11	0.30
Latvia	157,374	N	30	0.61
Lithuania	271,097	N	17	0.61
Luxembourg	173,380	N	64	3.55
Netherlands	817,300	N	22	0.70
Norway	206,299	Y	15	0.75
Poland	1,353,548	N	25	0.25
Portugal	746,322	Y	14	0.60
Romania	1,094,095	N	6	0.39
Slovakia	493,862	Y	7	0.60
Slovenia	326,572	N	19	0.90
Spain	1,045,937	Y	16	0.47
Sweden	1,370,970	N	23	1.10
Switzerland	401,086	N	20	0.71
United Kingdom	676,047	N	35	0.26
United States	803,121	N	9	-
*Total*	*23,145,668*			

Sources: (1) European Labour Force Survey and Current Population Survey.

(2)-(4) Eurostat (2006–2014); United States: data obtained upon request from the United States Census Bureau.

Notes:.

^1^ Non-response rates cover refusals and non-contacts.

^2^ Numbers pertain to the 2004–2014 interval because of previous limited availability. The non-response rate in the US pertains to the March 2000–2014 samples.

The analyses were restricted to women and men aged 25 to 54 and thereby exclude younger and older respondents who are, respectively, more likely to be in training contracts or early retirement schemes (Gebel and Giesecke, 2011). In accordance with the International Labour Organization, unemployed individuals are considered as those without work, who have been seeking work during the previous four weeks and are available to start working within the next two weeks. They are distinguished from employed persons who are in paid employment or self-employed (including those temporarily not at work in the reference period, e.g. because of maternity or parental leave) and inactive persons who neither work nor are looking for a job (e.g. homemakers).

A two-step multilevel approach (Gebel and Giesecke, 2011; Gebel and Giesecke, 2016) was used to evaluate the impact of economic fluctuations on inequality in unemployment and the moderating role of labour market institutions. The *first stage* employed logistic regression models to estimate the association of educational attainment with unemployment risk (controlling for gender and age) for each country and year for a sample of approximately 23 million observations. Since our main interest lies in understanding the dynamics of the education gap in unemployment across business cycle and its contextual moderators, the models remain parsimonious in the first stage and only include gender and age, while they omit other factors, such as sector, to preserve the sample's heterogeneity.

The main estimates shown in the paper focus on differences in employment status across active individuals. As robustness check, models were also estimated for employed versus unemployed/inactive. The shares of inactive persons differ greatly between countries, with particularly high rates of around 30% observed among women in Southern Europe (Italy, Greece, Spain), some Central and Eastern European countries (Hungary, Romania) as well as Ireland. Some inactive persons may be discouraged workers who would otherwise appear in the unemployment rankings, i.e., unemployed who have abandoned their job search—especially as the recession persists. Across all European countries and years, an average 10% of inactive men and 7% of inactive women stated that their reason for being inactive was that they believed no work was available. Between 2000 and 2014, inactivity increased from 7 to 13% (men) and from 4 to 9% (women), of which 19% of men and 9% of women were unemployed one year before.

Our education variables are defined according to the International Standard Classification of Education (ISCED): low (ISCED 0–2), medium (ISCED 3–4) and high education (ISCED 5–6). Table A1 shows the educational distribution by country. Across Europe, Southern European countries have a high share of low-educated persons, i.e., (less than) primary and lower secondary education, while the German-speaking as well as Central and Eastern European countries are characterised by large numbers of medium-educated individuals (i.e. upper secondary and post-secondary non-tertiary). The English-speaking and Nordic countries demonstrate the highest shares of tertiary educated individuals in the OECD (OECD, 2013a). The other Western European countries included (e.g. Belgium, the Netherlands, France) report relatively balanced shares across all three educational categories. While each country's educational distribution serves as important background information, it is beyond the scope of this paper to provide a detailed assessment of how different educational systems affect unemployment risk and how this changes over time and in connection to macro-economic conditions. Moreover, data restrictions (a variable is only available in EU-LFS from 2014) prevent us from distinguishing between the type of education within an ISCED category: vocational versus general. In countries with a strong educational emphasis on vocational training, such as German-speaking ones, vocational students acquire specialised skills and schools have a closer connection with employers than elsewhere (Breen, 2005). This ensures transparent educational credentials for employers, creating a tighter link between educational field and employment sector. In turn, this promotes an easier transition from school to the labour market than in countries that foster more general skills. During the Great Recession, this type of educational track has been proposed as an important instrument to combat (youth) unemployment. Germany, Austria and Poland are examples of countries with a strong vocational orientation, while a more generalised education is characteristic of Greece, Spain, the UK and the US (Andersen and Van De Werfhorst, 2010; OECD, 2013a).

The *second stage* used the estimated year–country coefficients of the unemployment gaps according to education as the dependent variable in macro-level models, which included country-level institutional and economic characteristics (and included both country and time fixed effects as well as their interactions with gender and age in some models, to control for characteristics not captured by other aggregate variables) (see table A2). The average marginal effects from the logistic models that represent the absolute difference in unemployment risk between low and highly educated individuals (as well as low and medium educated) were used as dependent variables in the macro-level models (for descriptive statistics, see table A3 and supplementary material figure S1).^1^ Average marginal effects were employed instead of odds ratios because prior research cautions against comparing odds ratios across models for different samples (Mood, 2010). Mean values for the average marginal effects were similar across gender and slightly larger for the younger group. In order to correct for the uncertainty associated with estimating the dependent variable in the first stage, standard errors were used from the first stage to adjust those in the second-stage models via a feasible generalised least squares approach (Lewis and Linzer, 2005). The main explanatory variables included both the change in GDP and the GDP level (divided by 1000) to account for the economic cycle as well as a set of labour market characteristics that comprise the one-year lagged shares of temporary contracts and public sector employees (including public administration, education, health/social work)^2^ among employed persons (see tables A2 and A3). The gender- and age-specific models used the share of temporary employment and public sector employment in the respective groups because of labour market stratification. In the models, the nonlinear effects of both temporary and public sector employment were also considered, and the role of temporary and public sector employment was examined separately in periods of either positive or negative (including zero) GDP growth. Finally, the EPL index regarding regular contracts (lagged by one year) was used to capture regulatory setting strictness, despite two caveats concerning this measure. First, it is missing from several countries for (almost) all years (Bulgaria, Cyprus, Lithuania, Latvia, Romania) or the earlier years (Estonia, Iceland, Luxembourg, Slovenia); second, it does not vary in nine countries throughout the period covered (Germany, Finland, Hungary, Iceland, Luxembourg, Norway, Poland, Switzerland and the US). The models that include the EPL index, hence, only draw on variation from a subset of the original countries. To account for different levels of selectivity in the education categories across countries and time periods, the main models were re-estimated to include the share of high educated in the country for each year. Results are robust to this inclusion and are provided in the supplementary material (see table S1).

## Descriptive analysis

**Figure 1.**
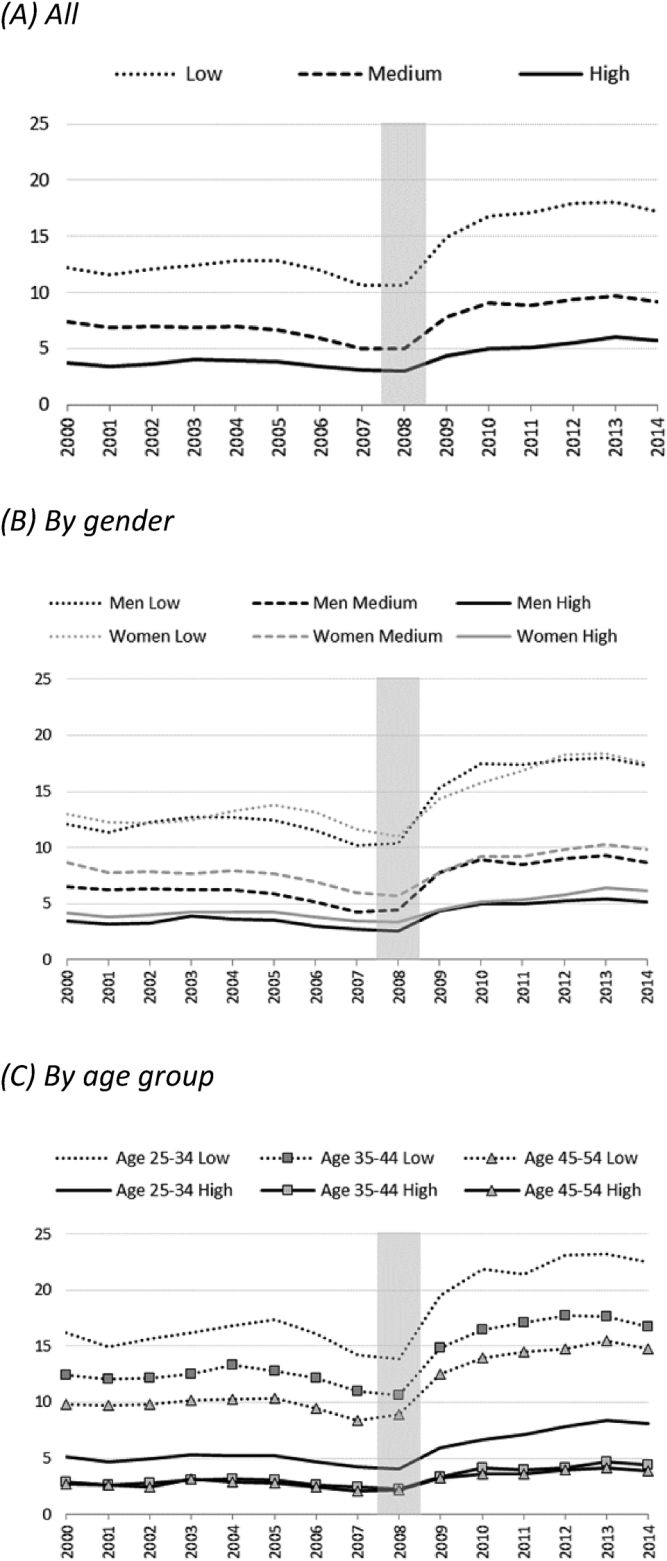
Unemployment rate by education (in %), 28 European countries and the United States. Note: Grey shading indicates the year 2008, the beginning of the Great Recession.

After high stability during the 2000–07 period, the unemployment rate increased steeply for all three educational groups between 2008 and 2010, especially among low-educated persons (Figure 1A). This increase was followed by a levelling-off in which unemployment prevalence stagnated across all three educational groups at a higher level than before the crisis. Additional analyses by gender reveal that the jump in unemployment in the years immediately after the recession (2009–10) was more pronounced among men than women, but the female unemployment rate surpassed the male rate again (as before the recession) in the following years (Figure 1B). This implies that the recession affected both men and women, but with some delay for the latter. Women's unemployment may have also been moderated by changes in activity rates. Concerning different age groups, the recession had the greatest effect on both low- and high-educated young persons (Figure 1C). Overall, differences between age groups were larger than differences by gender.

**Figure 2.**
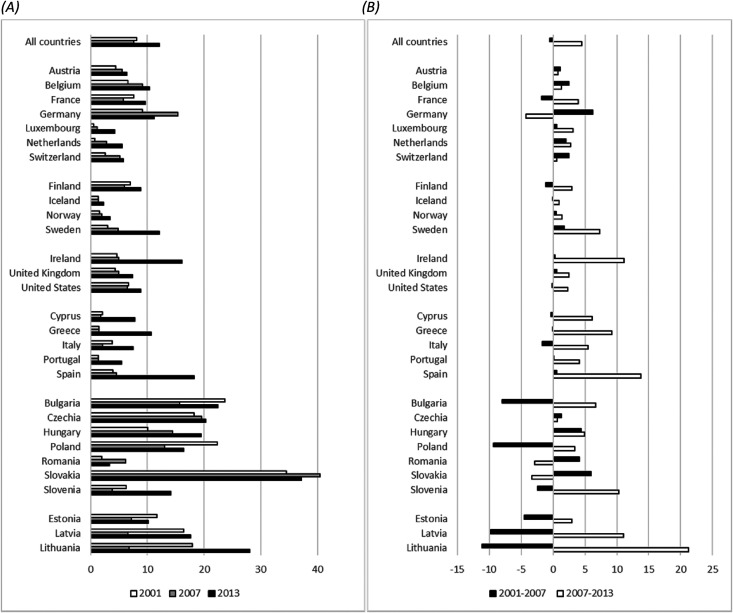
(A) Gap in the unemployment rate between low and highly educated persons (in percentage points) and (B) change in the unemployment rate gap between low and highly educated persons (in percentage points).

**Figure A1.**
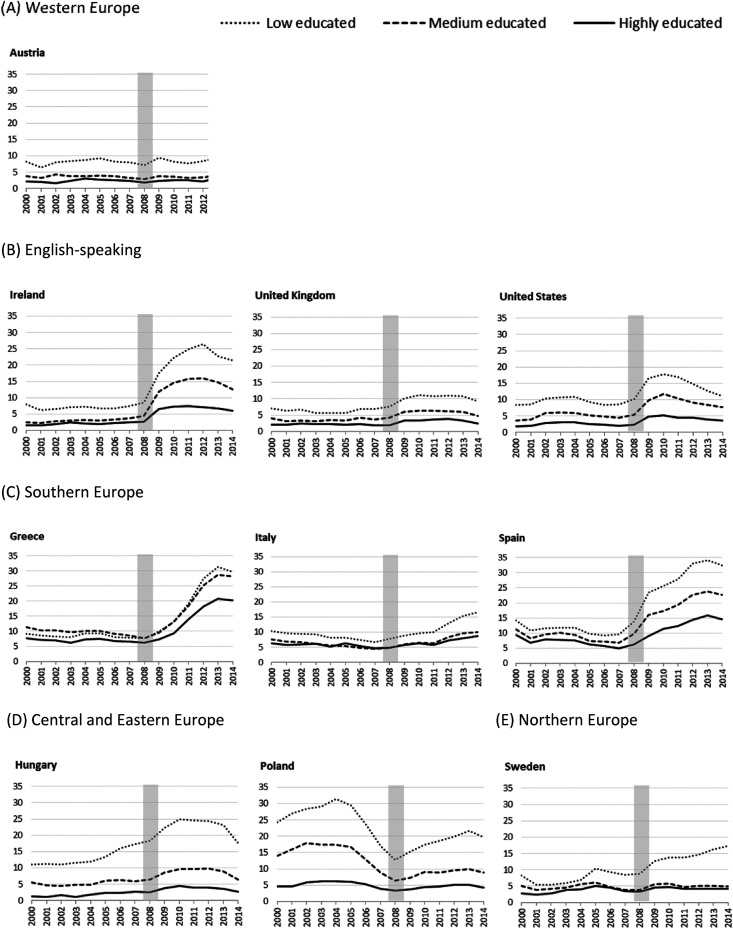
Unemployment rate by education (in %), selected countries.

Turning to individual countries, Figure 2 provides a summary of the unemployment trends for all 29 countries by showing, first, the absolute difference in the unemployment rate between low- and highly educated individuals in 2001, 2007 and 2013 (panel A) and, second, the absolute change in this gap for the two seven-year periods immediately before the crises (2001–07) and since the beginning of the recession (2007–13) (panel B) (see figure S2 for gap between low and medium educated persons). Unemployment trends by education in selected countries are depicted in the appendix (figure A1). Several distinct patterns appear in these data. During the first period, unemployment gaps were generally stable across Western Europe, as well as in the Nordic and the English-speaking countries, while they increased moderately in the second period. Germany and Ireland were the primary exceptions to those patterns: The German unemployment gap rose steeply in the pre-recession period, but has declined strongly since 2007. Conversely, Ireland faced one of the greatest increases in the unemployment gap of all the countries at the onset of the recession. In Southern Europe, the unemployment gap by education was very stable before the recession, but has climbed sharply since then (with the exception of Portugal, which showed a much fainter increase). Results are more mixed for Central and Eastern Europe, where patterns were heterogeneous prior to the recession. However, since the crisis, most of these countries (except for Romania and Slovakia) experienced a(n) (continued) increase in the unemployment gap. The Great Recession particularly affected the least educated in the Baltic countries.

## Macro-level analysis

Table 2A shows estimates from macro-level regressions on the determinants of the education gap in unemployment between low- and high-educated persons. To study gaps at the lower end of the educational distribution, table 2B includes similar models for the gap between low and medium educated. Table 3 depicts models, first, with interactions between contextual variables for different age groups and, second, with similar interactions for gender. These interaction terms measure whether the difference between the reference group (either young or men) and each of the other groups is significant. Table 4 adds the EPL index and also provides age and gender interactions. Complete tables with standard errors are available upon request from the authors.

**Table 2.** Macro-level determinants of the education gap in unemployment vs. employment.

	Model 1	Model 2	Model 3	Model 4	Model 5	Model 6	Model 7
(A) between low and high levels of education							
GDP change	−0.0038***	−0.0028***	−0.0032***	−0.0030***	−0.0032***	−0.0030***	−0.0033***
GDP level	−0.0031***	−0.0025**	−0.0041***	−0.0036***	−0.0040***	−0.0038***	−0.0036***
Temporary employment	−0.0037***	−0.0029***	−0.0041***	−0.0042***	0.0036*		−0.0047***
GDP change*Temporary employment		−0.0002*					
Public sector			0.0061***	0.0244***	0.0047***	0.0059***	
Public sector squared				−0.0004***			
Temporary employment squared					−0.0003***		
Positive temporary employment						−0.0041***	
Negative temporary employment						−0.0036***	
Positive public sector							0.0040***
Negative public sector							0.0041***
Constant	0.1876***	0.1615***	0.0948*	−0.1620*	0.0974**	0.0867*	0.1252***
Number of observations	435	435	434	434	434	434	435
R-squared	0.914	0.915	0.919	0.922	0.924	0.920	0.918
	Model 1	Model 2	Model 3	Model 4	Model 5	Model 6	Model 7
(B) between low and medium levels of education							
GDP change	−0.0010*	−0.0002	−0.0008*	−0.0007#	−0.0008*	−0.0007#	−0.0008#
GDP level	−0.0014*	−0.0010#	−0.0016**	−0.0013*	−0.0016**	−0.0014*	−0.0015**
Temporary employment	−0.0021***	−0.0015**	−0.0022***	−0.0022***	0.0019		−0.0023***
GDP change*Temporary employment		−0.0001*					
Public sector			0.0012	0.0144***	0.0005	0.0011	
Public sector squared				−0.0003***			
Temporary employment squared					−0.0002***		
Positive temporary employment						−0.0022***	
Negative temporary employment						−0.0017**	
Positive public sector							0.0009
Negative public sector							0.0009
Constant	0.0962***	0.0776**	0.0775**	−0.1061*	0.0788**	0.0705**	0.0819**
Number of observations	435	435	434	434	434	434	435
R-squared	0.9288	0.9298	0.9291	0.9317	0.9316	0.9296	0.9292

Notes: Significance levels: *** p < 0.001; ** p < 0.01; * p < 0.05; # p < 0.10. All variables lagged by 1 year. Macro-level regressions (level 2) include country and year fixed effects (not shown). The dependent variable is the gap in the likelihood of unemployment between low- and high-educated persons (or low- and medium-educated persons, respectively) estimated with logistic regression models for each country and year separately (level 1).

**Table 3.** Macro-level determinants of the education gap in unemployment vs. employment between low and high levels of education, interactions with age group and gender.

	Model 1	Model 2	Model 3	Model 4	Model 5		Model 1	Model 2	Model 3	Model 4	Model 5
Middle (35–44 years)	0.0044	−0.0237	−0.0172	−0.0047	−0.0453	Woman	−0.0994#	−0.0467	−0.1061#	0.0701	−0.1082*
Old (45–54 years)	0.0064	−0.0261	−0.0372	0.0392	−0.0475						
GDP change	−0.0045***	−0.0042***	−0.0042***	−0.0041***	−0.0041***	GDP change	−0.0045***	−0.0024**	−0.0042***	−0.0043***	−0.0041***
X Middle	0.0015	0.0026*	0.0013	0.0013	0.0013	X Woman	0.0017*	−0.0006	0.0016#	0.0017#	0.0015#
X Old	0.0005	0.0023#	0.0007	0.0006	0.0007						
GDP level	−0.0025*	−0.0022*	−0.0045***	−0.0037***	−0.0049***	GDP level	−0.0040***	−0.0030**	−0.0049***	−0.0039***	−0.0047***
X Middle	−0.0009	−0.0003	0.0002	−0.0005	0.0004	X Woman	0.0022#	0.0011	0.0029*	0.0019	0.0026*
X Old	−0.0014	−0.0007	0.0006	0.0000	0.0008						
Temporary employment	−0.0018**	−0.0016*	−0.0024***	−0.0023***	0.0046**	Temporary employment	−0.0032***	−0.0012	−0.0035***	−0.0038***	0.0071***
X Middle	−0.0037***	−0.0027*	−0.0032**	−0.0035**	0.0014	X Woman	−0.0005	−0.0026*	−0.0002	0.0000	−0.0038
X Old	−0.0016	0.0002	−0.0014	−0.0014	0.0011						
GDP change* Temporary employment		0.0000				GDP change* Temporary employment		−0.0004***			
X Middle		−0.0002*				X Woman		0.0004***			
X Old		−0.0004**									
Public sector			0.0058***	0.0160***	0.0045***	Public sector			0.0047***	0.0257***	0.0031*
X Middle			−0.0023	−0.0014	−0.0011	X Woman			−0.0030*	−0.0218***	−0.0018
X Old			−0.0027*	−0.0076	−0.0019						
Public sector squared				−0.0002**		Public sector squared				−0.0006***	
X Middle				0.0000		X Woman				0.0006***	
X Old				0.0001							
Temporary employment squared					−0.0002***	Temporary employment squared					−0.0004***
X Middle					−0.0003***	X Woman					0.0002*
X Old					−0.0004*						
Constant	0.1944***	0.1841***	0.1688***	0.0164	0.1738***	Constant	0.2319***	0.1840***	0.1972***	−0.0193	0.1876***
Number of observations	1305	1305	1302	1302	1302	Number of observations	870	870	868	868	868
R-squared	0.8940	0.8956	0.8991	0.9004	0.9049	R-squared	0.9025	0.9044	0.9043	0.9059	0.9116

Notes: See table 2. Public sector and temporary employment shares are specific to the group employed in the estimation. The models include country and year fixed effects, as well as interactions of country and year with age group and gender, respectively (not shown). We performed joint tests of differences in temporary and public employment between age groups and found that all differences are statistically significant at p < 0.001. Joint tests of differences in temporary and public employment between men and women showed that all differences are statistically significant at p < 0.001 (except for M5 public sector, which was significant at p < 0.01).

**Table 4.** Macro-level determinants of the education gap in unemployment vs. employment between low and high levels of education (including EPL regular).

	All	Age groups	Gender
Woman			−0.0609
Middle (35–44 years)		0.1575#	
Old (45–54 years)		0.0919	
GDP change	−0.0029***	−0.0040***	−0.0044***
X Middle		0.0030*	
X Old		−0.0001	
X Woman			0.0028*
GDP level	−0.0063***	−0.0084***	−0.0071***
X Middle		0.0008	
X Old		0.0024	
X Woman			0.0017
Temporary employment	−0.0039***	−0.0026***	−0.0036***
X Middle		−0.0048***	
X Old		−0.0006	
X Woman			−0.0002
Public sector	0.0050***	0.0073***	0.0040**
X Middle		−0.0047**	
X Old		−0.0056***	
X Woman			−0.0030#
EPL regular	0.0175#	0.0582***	0.0185
X Middle		−0.0581***	
X Old		−0.0531**	
X Woman			0.0010
Constant	0.1651**	0.1499*	0.2524***
Number of observations	323	969	646
R-squared	0.941	0.9348	0.9353

Notes: See table 2. Public sector and temporary employment shares are specific to the group employed in the estimation. Included are country and year fixed effects as well as interactions of country and year with age group and gender, respectively (not shown). Standard errors in second stage not corrected. We performed joint tests of differences in EPL regular between age groups and found that all differences are statistically significant at p < 0.001. Differences between men and women are not significant (p = 0.0608).

Results in table 2 reveal that, within countries, larger drops in GDP growth and lower GDP level (lagged one year) are both associated with a significantly higher absolute education gap in unemployment between low- and high-educated persons (model 1). This aligns with the expectation that recessions tend to affect lower-educated employees most profoundly. Depending on GDP level, periods of relatively large changes to GDP are associated with greater changes in the educational gap. Those periods may reflect when more job creation or destruction occurs. Table 2B shows models for the gap between medium and low educated and demonstrates that the business cycle plays a more subdued role (in size and significance) than it does for explaining the gap between high and low educated, most likely because it affects both education groups at the lower end of the distribution quite strongly. Models for the gap between medium and low educated that contain interactions with gender and age are presented in the supplementary material (table S2).

As predicted by *hypothesis 1a*, changes in GDP growth and in GDP level within countries are generally associated with larger oscillations in the education gap in unemployment among men than among women (table 3) and the interaction coefficient of women and changes in GDP is significant (model 1).^3^ GDP change is significant for all age groups (model 1, table 3); but, as expected, it has the largest effect for the youngest age group (even though the differences are only weakly significant in model 2). However, the implied association for GDP level is similar across age groups. Therefore, these results partly support *hypothesis 1b*, which posited that the recession effect would have a larger impact on young, less-educated workers.

Additional specifications (not included in this paper) were used to estimate whether periods of relatively large GDP increases were associated with larger shifts in the educational unemployment gap compared to periods of large GDP decreases. Interestingly, for old and middle-aged individuals, relatively large drops in GDP (likely associated with job destruction) widened the education gap the most Conversely, periods of large economic gains (likely concurrent with job creation) seem to close the education gap the most for young individuals. For the entire sample, large negative changes seem to matter most. One possible explanatory mechanism for this finding is that only periods of intense job restructuring threaten the relatively more-protected jobs of older workers. On the other hand, access to employment for low skilled, young workers chiefly opens up in periods of strong job growth.

With regard to temporary employment, a lower prevalence of temporary employment within a country was associated with larger educational disparities in terms of unemployment—both between high and low educated as well as between middle and low educated (tables 2A & B, model 1). Temporary employment serves as a bridge to the labour market for low-educated individuals, wherein temporary contracts help close the unemployment gap when they (re)enter the workforce (supporting *hypothesis 2*). While the results uncovered minor differences between men and women, the education gap grew (significantly) the most among middle-aged persons (35–44 years) when temporary employment declined (table 3). One possible explanation is that low-educated persons in this age group disproportionally hold temporary jobs, while the prevalence of temporary work is larger and more equally spread among the youngest across educational attainment. The negative coefficient of the interaction between GDP growth and temporary contracts (tables 2 and 3, model 2) also reveals that (except among women and the young), within a country, the low educated benefit more from the availability of temporary contracts in times of higher GDP growth, while the opposite is true in periods of large GDP drops.

Coefficients in model 3 for within-country changes in the size of the public sector (mostly downsizing during this period) are in line with *hypothesis 3* (though the impact is quite moderate for differences between middle and low educated). Public sector jobs have greater stability and protection during times of economic crisis than private sector ones and may shield the highly educated, who are overrepresented in these positions, from becoming unemployed. The effect is weaker among women than among men, arguably because their education gradient in public employment is less pronounced and because a larger share of women than men are employed in the public sector across all countries (see table A3). Further, the education gap in unemployment tends to be larger for younger persons when the public sector is increasing (or shrinking less) within a country, because only the relatively more-educated young people can access or retain jobs that otherwise tend to be held by older insiders.

Models 4 and 5 (tables 2 and 3) explored nonlinearities in the association of either temporary employment or public sector employment and the educational gap. Model 4 revealed that temporary employment plays only a small role in closing the educational gap until the temporary share moves well above the mean of the panel (around 8.4%). This is particularly relevant for countries with relatively high shares of temporary employment resulting from the deregulation that started in the early 1990s (e.g. Spain). Model 5 coefficients imply that expansions to public sector employment are associated with increases in the gap, although the implied changes are very minor for countries whose public sectors are already large, such as the Nordic countries. It is this rise from low- to middle-sized public sectors that helps open up the unemployment gap by offering more stable employment opportunities to relatively more-educated. The opposite is true during downsizing.

As a follow-up sensitivity analysis, model 3 was estimated separately for two geographic clusters (see appendix table A4). Cluster 1 comprises Western, English-speaking and Nordic countries and cluster 2 comprises Southern, CEE and Baltic countries. The negative association with temporary employment only holds for cluster 2, where temporary employment is generally more prevalent and where the negative education gradient therein is more marked. Conversely, a positive association with temporary employment was observed in cluster 1, which has a negligible gradient; the education gradient in temporary employment is less evident in these countries and was positive until 2005. Also consistent with the nonlinear results from model 5, the coefficient for the public sector in table A4 is only significant in cluster 2 and is much larger for countries that demonstrate relatively smaller sectors and likely have more educational selection.

Coefficients for both temporary and public employment were estimated separately during periods of growth versus periods of contraction to see if any asymmetries exist for the relevance of employment composition along the business cycle and found no differences (models 6 and 7, table 2).

Within the set of countries and years for which data was available (table 4), a high regular contract EPL indicator (higher protection of regular workers) is associated with a larger education gap in a country's unemployment, especially among younger persons and—to some extent—women, as proposed by *hypothesis 4*. The relationship is even stronger for women in models that consider both unemployed and inactive versus employed (results available upon request).^4^

## Concluding discussion

We studied changes in the education gap in unemployment prevalence during and since the Great Recession in 28 European countries and the US to ascertain whether economic inequality in the form of larger unemployment gaps increased in these societies (Van de Werfhorst and Salverda, 2012). A central result is that the Great Recession ended the rather long period of stability in the education gap in unemployment across European workers, which lasted throughout the 1990s and up until 2007 (see Gebel and Giesecke, 2011). The findings show that the absolute education gap in unemployment widened in almost all of the countries from 2009 onward. This means that unemployment inequality rose and education became an increasingly important determinant of a person's economic standing. Moreover, the rise in unemployment during and immediately after the recession was especially pronounced among low-educated men. This result is consistent with the notion of a “male recession,” which is expected to entail negative consequences for livelihood and relationship quality given that men still tend to be the main breadwinners—and even more so among low-educated families. However, after this initial spike in male unemployment, women's unemployment also rose. For the US, Folbre described this development as “his recession, becoming hers” (Folbre, 2011) when public spending shrank for occupations such as teachers or social workers. Across age groups, young persons—even some highly educated—were most intensely affected by the recession (Bell and Blanchflower, 2011a). Our study focused on unemployment and only briefly touched upon the issue of labour market discouragement. However, the social and economic consequences (e.g. on well-being) experienced by those who drop out of the labour force might be as severe—or worse—compared to the unemployed who still have the hope of finding a job. In the US, a higher share of older unemployed persons (compared to younger ones) left the workforce during the Great Recession, as it was much harder to regain employment (Van Horn et al., 2011). To some extent, this potentially moderated the observed gap between high- and low-educated older individuals. Future research could explore the developments in labour market discouragement in detail.

Macro-level models were used to analyse the interaction between labour market institutions and business cycle shocks following previous work in this area (Bertola et al., 2007; DiPrete et al., 2006; Gebel and Giesecke, 2011). We documented a negative relationship within countries between both GDP level and GDP growth and the absolute gap in unemployment between low- and highly educated individuals. Additionally, we showed how institutional labour market characteristics moderated (or did not) the effect of these business cycle oscillations. Within countries, the unemployment gap was smaller in periods with a high prevalence of temporary employment, which gave the lower educated easier (re)entry to the labour market through these jobs. Thus, after recession periods where the proportion of temporary workers drops (which affects the low educated relatively more) (Holmlund and Storrie, 2002), the low educated benefit more from temporary job creation as the economy recovers. Our nonlinear estimates of the impact of the temporary employment size imply this result holds chiefly for Southern and Central and Eastern European countries, where temporary employment expanded after deregulation (Dolado et al., 2002) and is, on average, more frequent and associated with a stronger educational gradient. However, while temporary employment might be a way to re-enter the workforce, previous research also characterised it as an insecure and economically precarious form of employment that often entails fewer social benefits (e.g. OECD, 2002; Van Lancker, 2012). In some countries, the growth of temporary employment represents a shift away from standard employment.

Moreover, we find that an increase (or more moderate downsizing) in public sector employment slightly amplifies the skills divide, because highly educated persons tend to be better shielded from unemployment in permanent positions as public servants. The implied association is minor for women, as they are generally more likely to be public sector employees at all educational levels. In both Northern Europe and the US, the public sector has traditionally opened up opportunities for least-skilled and minority women (King, 1993). Finally, consistent with previous work that shows how highly regulated labour markets in Europe especially penalised young workers and women during the 1990s and early 2000s (Bertola et al., 2007; Gebel and Giesecke, 2011), we find that a stricter regular contract EPL within countries is related to a larger education gap in unemployment, particularly among the young (Gebel and Giesecke, 2011).

While the data showed a decline in unemployment across European countries from 2014 and from 2010 for the US, the structural component of unemployment (as opposed to the cyclical component) increased in some European countries, which prevented the unemployment rate from quickly declining to pre-recession levels (OECD, 2013b) and led to an increase in the incidence of long-term unemployment (particularly concentrated among low-educated persons) and a consolidation of educational inequalities. Under this scenario, educational disparities in life satisfaction, health and political participation (Gallie et al., 2001; McKee-Ryan et al., 2005; Paul and Moser, 2009) are anticipated to grow, and social cohesion between educational groups and the workings of the welfare state in the studied countries are expected to come under strain.

## Supplemental Material

sj-docx-1-asj-10.1177_00016993221083226 - Supplemental material for Growing inequality during the Great Recession: Labour market institutions and the education gap in unemployment across Europe and in the United States Click here for additional data file. Supplemental material, sj-docx-1-asj-10.1177_00016993221083226 for Growing inequality during the Great Recession: Labour market institutions and the education gap in unemployment across Europe and in the United States by Caroline Berghammer and Alicia Adserà in Acta Sociologica

## Notes

1. Few outliers (due to low case numbers) were excluded.

2. There is not a single data source that offers comparable information on the share of public employment (Tepe, 2009). As a robustness check, we also used an additional measure from the OECD publication “Government at a Glance,” which is, however, only available for a limited number of countries and years.

3. In models studying the education gap in the likelihood of being unemployed or inactive versus employed, shown in table A5 in appendix, coefficients for both GDP indicators are significant for women. The latter may be more likely to adjust to shocks via their participation in the labour market than men.

4. In supplementary models in table S3, we interacted the changes in GDP with the EPL level. We find that a strong EPL moderates the extent to which the educational gap increases during recessions.

## APPENDIX

**Table A1.** Educational distribution (in %), 2000 and 2014.

		2000	2014		2000	2014
Austria	Low	20	14	Lithuania	8	7
	Medium	64	54		48	54
	High	15	32		44	39
Belgium	Low	37	22	Luxembourg	36	16
	Medium	34	38		44	35
	High	30	40		19	49
Bulgaria	Low	27	17	Netherlands	31	20
	Medium	53	54		44	43
	High	19	29		25	37
Cyprus	Low	33	18	Norway	11	17
	Medium	39	38		55	37
	High	28	44		34	46
Czechia	Low	12	5	Poland	16	7
	Medium	77	71		73	61
	High	12	24		12	32
Estonia	Low	10	9	Portugal	77	50
	Medium	61	53		12	25
	High	29	38		10	24
Finland	Low	21	11	Romania	23	24
	Medium	44	45		68	58
	High	35	44		10	18
France	Low	34	19	Slovakia	12	7
	Medium	43	44		77	71
	High	23	37		11	22
Germany	Low	16	13	Slovenia	22	11
	Medium	59	60		62	57
	High	25	28		17	32
Greece	Low	42	26	Spain	57	38
	Medium	39	44		18	23
	High	19	30		25	39
Hungary	Low	23	15	Sweden	19	13
	Medium	62	60		49	45
	High	15	26		32	41
Iceland	Low	41	25	Switzerland	16	11
	Medium	33	35		59	46
	High	26	40		26	42
Ireland	Low	38	16	United Kingdom	35	21
	Medium	38	39		36	37
	High	23	45		29	42
Italy	Low	49	37	United States	11	11
	Medium	40	45		51	44
	High	11	18		38	45
Latvia	Low	12	11			
	Medium	69	57			
	High	19	33			

**Table A2.** Summary statistics on macro-level variables.

Country	GDP change (in %)^a^			GDP level^b^			Temporary employment (in %)^c^			Public sector employment (in %)^d^			Employment protection legislation – regular^e^		
	2000	2014	mean	2000	2014	mean	2000	2014	mean	2000	2014	mean	2000	2014	mean
Austria	3.4	0.4	1.5	42001	47922	45696	3.6	4.9	4.2	22.3	24.6	23.5	2.8	2.4	2.5
Belgium	3.6	1.3	1.5	40170	44677	43150	6.9	7.2	6.7	32.5	32.5	32.5	1.8	1.9	1.9
Bulgaria	5.0	1.5	3.6	3955	7300	5978	6.6	5.1	5.2	21.3	19.1	20.1	–	–	–
Cyprus	6.0	−1.3	2.0	27318	27046	29654	9.3	18.8	13.6	19.7	23.1	20.3	–	–	–
Czechia	4.3	2.0	2.6	14807	20344	18405	5.2	7.4	6.2	19.5	20.1	19.8	3.3	2.9	3.2
Estonia	10.6	2.9	4.2	10108	17453	14616	2.2	2.5	2.4	19.2	22.6	21.4	–	1.8	2.1
Finland	5.6	−0.6	1.5	40450	45239	45166	13.3	11.3	11.4	27.7	29.5	28.7	2.3	2.2	2.2
France	3.9	0.2	1.3	38461	41375	40365	11.6	13.0	11.0	28.4	33.1	30.5	2.3	2.4	2.4
Germany	3.0	1.6	1.2	37998	45023	41016	7.3	9.0	8.2	24.1	26.0	25.3	2.7	2.7	2.7
Greece	4.2	0.7	0.2	23275	22566	26046	12.1	11.9	11.1	20.8	23.3	22.1	2.8	2.1	2.7
Hungary	4.2	3.7	2.0	10490	14119	12795	6.1	12.5	8.6	22.3	25.6	23.2	2.0	1.6	2.0
Iceland	4.6	2.2	2.8	36555	44776	41949	3.8	10.5	7.3	25.3	28.4	28.6	–	1.7	1.7
Ireland	10.2	5.2	3.4	42945	54053	49444	3.0	6.8	4.7	21.7	27.7	25.5	1.4	1.4	1.4
Italy	3.7	−0.4	0.2	36181	33616	36277	8.8	12.8	10.9	25.0	20.7	22.5	2.8	2.7	2.8
Latvia	5.4	2.4	4.1	6935	13759	11090	5.9	2.9	5.9	23.3	23.2	23.4	–	2.7	2.7
Lithuania	3.8	3.0	4.4	6934	14936	11251	3.1	2.2	3.7	23.1	23.6	23.2	–	–	–
Luxembourg	8.2	5.8	3.2	93463	107153	102348	2.4	5.6	4.1	26.0	34.6	31.2	–	2.3	2.3
Netherlands	4.2	1.0	1.2	46133	50497	49085	9.5	14.8	10.8	31.4	35.2	34.3	2.9	2.8	2.9
Norway	3.2	2.0	1.7	81710	89275	87346	6.8	5.9	6.7	34.3	36.6	36.3	2.3	2.3	2.3
Poland	4.3	3.3	3.6	8526	14091	11165	4.6	25.6	19.7	20.3	21.7	21.1	2.2	2.2	2.2
Portugal	3.8	0.9	0.3	21513	21533	21962	15.8	19.0	18.0	21.2	27.1	23.9	4.6	3.2	4.3
Romania	2.4	2.8	3.6	4910	9227	7422	2.0	1.2	1.4	13.9	13.2	14.9	–	2.6	2.6
Slovakia	1.2	2.5	3.9	10297	18004	14579	2.9	7.1	4.3	23.9	23.8	22.6	2.5	1.8	2.2
Slovenia	4.2	3.0	2.1	18571	23224	22218	8.7	12.7	11.4	18.6	22.3	20.8	–	–	–
Spain	5.3	1.4	1.6	28335	29496	30364	28.6	23.4	25.7	20.3	24.1	22.5	2.4	2.0	2.3
Sweden	4.7	2.6	2.2	44694	53562	50233	11.1	12.6	11.6	33.7	35.1	34.4	2.7	2.6	2.6
Switzerland	3.9	2.4	2.0	67808	76411	72270	4.2	5.9	5.9	27.6	27.1	26.0	1.6	1.6	1.6
United Kingdom	3.8	2.9	1.9	35577	40909	38976	5.3	3.9	4.2	28.8	32.3	31.5	1.1	1.1	1.2
United States	4.1	2.4	1.9	45056	50872	48147	2.9	3.2	3.1	19.5	23.5	22.3	0.3	0.3	0.3

Notes: ^a^ Data are from the World Bank (2016): GDP growth (annual %). ^b^ Data are from the World Bank (2016): GDP per capita (constant 2010 $US). ^c^ Own calculations based on the Labour Force Surveys and Current Population Surveys. ^d^ Own calculations based on the Labour Force Surveys and Current Population Surveys. Public sector employment includes public administration, education and health/social work. ^e^ Data are from the OECD (2016). They pertain to 2013, because no data for 2014 available.

**Table A3.** Summary statistics on average marginal effects (difference in likelihood of unemployment) and share of public and temporary employment.

	Mean	Std.dev.	Min.	Max.
Unemployment Gap				
(a) between low and highly educated				
All	0.100	0.084	−0.004	0.480
Men	0.099	0.093	−0.006	0.542
Women	0.105	0.083	−0.008	0.452
Young (25–34 years)	0.126	0.112	−0.021	0.703
Middle (35–44 years)	0.106	0.094	−0.004	0.536
Old (45–54 years)	0.083	0.075	−0.014	0.396
(b) between low and medium educated				
All	0.067	0.065	−0.015	0.390
Men	0.070	0.074	−0.006	0.460
Women	0.068	0.068	−0.046	0.343
Young (25–34 years)	0.094	0.090	−0.025	0.615
Middle (35–44 years)	0.071	0.075	−0.022	0.445
Old (45–54 years)	0.050	0.054	−0.022	0.286
Public employment in %				
All	25.1	5.3	13.8	37.6
Men	14.8	3.4	8.7	27.6
Women	36.5	8.7	17.4	57.3
Young (25–34 years)	22.2	5.5	12.2	40.5
Middle (35–44 years)	25.0	5.0	12.8	38.6
Old (45–54 years)	27.7	6.2	13.5	41.6
Temporary employment in %				
All	8.4	5.7	0.8	28.9
Men	7.5	5.3	0.8	27.8
Women	9.4	6.4	0.7	32.1
Young (25–34 years)	12.6	8.8	1.0	42.3
Middle (35–44 years)	7.4	5.1	0.5	26.1
Old (45–54 years)	5.6	3.5	0.6	18.5

**Table A4.** Macro-level determinants of the education gap in unemployment vs. employment between low and high levels of education.

	Cluster 1	Cluster 2
GDP change	−0.0026**	−0.0027***
GDP level	−0.0029**	−0.0066***
Temporary employment	0.0049***	−0.0065***
Public sector	0.0004	0.0090***
Number of observations	210	224
R-squared	0.845	0.934

Notes: See table 2. Cluster 1: Western European, English-speaking, Nordic countries. Cluster 2: Southern European, CEE, Baltic countries.

**Table A5.** Macro-level determinants of the education gap in unemployment/inactive vs. employment between low and high levels of education.

	All	Age groups	Gender
Middle (35–44 years)		0.0674	
Old (45–54 years)		0.1705*	
Woman			−0.1532*
GDP change	−0.0008	−0.0023***	−0.0026***
X Middle		0.0031**	
X Old		0.0009	
X Woman			0.0032***
GDP level	−0.0017*	−0.0008	−0.0053***
X Middle		−0.0029	
X Old		−0.0013	
X Woman			0.0068***
Temporary employment	−0.0031***	−0.0013	−0.0038***
X Middle		−0.0040**	
X Old		−0.0050***	
X Woman			0.0008
Public sector	0.0029**	0.0051***	0.0049***
X Middle		−0.0009	
X Old		−0.0062***	
X Woman			−0.0037*
Constant	0.1968***	0.1532**	0.2977***
Number of observations	434	1302	868
R-squared	0.9377	0.9111	0.9422

Notes: See table 2. Public sector and temporary employment shares are specific to the group employed in the estimation.
