# Supplementary material for: Growing inequality during the Great Recession: Labour market institutions and the education gap in unemployment across Europe and in the United States
Source: Acta Sociol. 2022 Mar 28;65(4):374–97. doi: 10.1177/00016993221083226 (PMC9536003; doi:10.1177/00016993221083226)

**Supplementary material**

**Table S1.** Macro-level determinants of the education-gap in unemployment vs. employment between low and high levels of education – controlling for level of education

|  | Model 1 | Model 2 | Model 3 | Model 4 | Model 5 |
| --- | --- | --- | --- | --- | --- |
| GDP change | -0.0035*** | -0.0026*** | -0.0031*** | -0.0029*** | -0.0032*** |
|  | (0.001) | (0.001) | (0.001) | (0.001) | (0.001) |
| GDP level | -0.0038*** | -0.0032** | -0.0043*** | -0.0038*** | -0.0041*** |
|  | (0.001) | (0.001) | (0.001) | (0.001) | (0.001) |
| Temporary employment | -0.0042*** | -0.0034*** | -0.0043*** | -0.0044*** | 0.0033# |
|  | (0.001) | (0.001) | (0.001) | (0.001) | (0.002) |
| GDP change*Temporary employment |  | -0.0002* |  |  |  |
|  |  | (0.000) |  |  |  |
| Public sector |  |  | 0.0057*** | 0.0246*** | 0.0044*** |
|  |  |  | (0.001) | (0.005) | (0.001) |
| Public sector squared |  |  |  | -0.0004*** |  |
|  |  |  |  | (0.000) |  |
| Temporary employment squared |  |  |  |  | -0.0003*** |
|  |  |  |  |  | (0.000) |
| **Share of highly educated** | **0.0019**** | **0.0018**** | **0.0008** | **0.0010** | **0.0006** |
|  | **(0.001)** | **(0.001)** | **(0.001)** | **(0.001)** | **(0.001)** |
| Number of observations | 435 | 435 | 434 | 434 | 434 |
| R-squared | 0.914 | 0.915 | 0.919 | 0.922 | 0.924 |

Notes: Significance levels: *** p<0.001; ** p<0.01; * p<0.05; # p<0.10; standard errors in parentheses. All variables lagged by 1 year. Macro-level regressions (level 2) include country and year fixed effects (not shown). The dependent variable is the gap in the likelihood of unemployment between low and high educated persons estimated with logistic regression models for each country and year separately (level 1).

**Table S2.** Macro-level determinants of the education-gap in unemployment vs. employment between low and medium levels of education, interactions with age group and gender

|  | Model 1 | Model 2 | Model 3 | Model 4 | Model 5 |  | Model 1 | Model 2 | Model 3 | Model 4 | Model 5 |
| --- | --- | --- | --- | --- | --- | --- | --- | --- | --- | --- | --- |
|  |  |  |  |  |  |  |  |  |  |  |  |
| Middle (35-44 years) | -0.0589 | -0.0536 | -0.0609 | -0.0220 | -0.0744# | Woman | -0.1108** | -0.0632# | -0.0767# | -0.0077 | -0.0822* |
|  | (0.042) | (0.044) | (0.043) | (0.077) | (0.042) |  | (0.037) | (0.038) | (0.039) | (0.066) | (0.039) |
| Old (45-54 years) | -0.0628 | -0.0631 | -0.0717 | -0.0209 | -0.0776# |  |  |  |  |  |  |
|  | (0.041) | (0.043) | (0.044) | (0.067) | (0.043) |  |  |  |  |  |  |
| GDP change | -0.0017*** | -0.0010# | -0.0015** | -0.0015** | -0.0015** | GDP change | -0.0016*** | 0.0001 | -0.0014*** | -0.0015*** | -0.0014** |
|  | (0.000) | (0.001) | (0.000) | (0.000) | (0.000) |  | (0.000) | (0.001) | (0.000) | (0.000) | (0.000) |
| X Middle | 0.0016* | 0.0016# | 0.0014* | 0.0014* | 0.0014* | X Woman | 0.0017** | -0.0004 | 0.0014* | 0.0014* | 0.0014* |
|  | (0.001 | (0.001) | (0.001) | (0.001) | (0.001) |  | (0.001) | (0.001) | (0.001) | (0.001) | (0.001) |
| X Old | 0.0007 | 0.0012 | 0.0007 | 0.0006 | 0.0007 |  |  |  |  |  |  |
|  | (0.001) | (0.001) | (0.001) | (0.001) | (0.001) |  |  |  |  |  |  |
| GDP level | -0.0018* | -0.0013# | -0.0028*** | -0.0023** | -0.0030*** | GDP level | -0.0024*** | -0.0016** | -0.0030*** | -0.0024*** | -0.0029*** |
|  | (0.001) | (0.001) | (0.001) | (0.001) | (0.001) |  | (0.001) | (0.001) | (0.001) | (0.001) | (0.001) |
| X Middle | 0.0005 | 0.0004 | 0.0012 | 0.0007 | 0.0013 | X Woman | 0.0024** | 0.0013 | 0.0030*** | 0.0026** | 0.0029*** |
|  | (0.001) | (0.001) | (0.001) | (0.001) | (0.001) |  | (0.001) | (0.001) | (0.001) | (0.001) | (0.001) |
| X Old | 0.0002 | 0.0002 | 0.0012 | 0.0007 | 0.0013 |  |  |  |  |  |  |
|  | (0.001) | (0.001) | (0.001) | (0.001) | (0.001) |  |  |  |  |  |  |
| Temporary employment | -0.0010* | -0.0006 | -0.0011* | -0.0011* | 0.0025* | Temporary employment | -0.0017*** | -0.0002 | -0.0018** | -0.0019*** | 0.0027* |
|  | (0.000) | (0.000) | (0.000) | (0.000) | (0.001) |  | (0.001) | (0.001) | (0.001) | (0.001) | (0.001) |
| X Middle | -0.0023** | -0.0022* | -0.0022* | -0.0024** | -0.0005 | X Woman | -0.0005 | -0.0023** | -0.0005 | -0.0003 | -0.0002 |
|  | (0.001) | (0.001) | (0.001) | (0.001) | (0.002) |  | (0.001) | (0.001) | (0.001) | (0.001) | (0.002) |
| X Old | -0.0007 | 0.0000 | -0.0008 | -0.0008 | 0.0001 |  |  |  |  |  |  |
|  | (0.001) | (0.001) | (0.001) | (0.001) | (0.002) |  |  |  |  |  |  |
| GDP change*Temporary employment |  | -0.0001* |  |  |  | GDP change*Temporary employment |  | -0.0003*** |  |  |  |
|  |  | (0.000) |  |  |  |  |  | (0.000) |  |  |  |
| X Middle |  | 0.0000 |  |  |  | X Woman |  | 0.0004*** |  |  |  |
|  |  | (0.000) |  |  |  |  |  | (0.000) |  |  |  |
| X Old |  | -0.0002 |  |  |  |  |  |  |  |  |  |
|  |  | (0.000) |  |  |  |  |  |  |  |  |  |
| Public sector |  |  | 0.0027*** | 0.0103*** | 0.0021** | Public sector |  |  | 0.0028** | 0.0145*** | 0.0021* |
|  |  |  | (0.001) | (0.002) | (0.001) |  |  |  | (0.001) | (0.004) | (0.001) |
| X Middle |  |  | -0.0017# | -0.0034 | -0.0011 | X Woman |  |  | -0.0034** | -0.0123** | -0.0030** |
|  |  |  | (0.001) | (0.005) | (0.001) |  |  |  | (0.001) | (0.005) | (0.001) |
| X Old |  |  | -0.0018# | -0.005 | -0.0014 |  |  |  |  |  |  |
|  |  |  | (0.001) | (0.004) | (0.001) |  |  |  |  |  |  |
| Public sector squared |  |  |  | -0.0002** |  | Public sector squared |  |  |  | -0.0003** |  |
|  |  |  |  | (0.000) |  |  |  |  |  | (0.000) |  |
| X Middle |  |  |  | 0.0000 |  | X Woman |  |  |  | 0.0003* |  |
|  |  |  |  | (0.000) |  |  |  |  |  | (0.000) |  |
| X Old |  |  |  | 0.0001 |  |  |  |  |  |  |  |
|  |  |  |  | (0.000) |  |  |  |  |  |  |  |
| Temporary employment squared |  |  |  |  | -0.0001*** | Temporary employment squared |  |  |  |  | -0.0002*** |
|  |  |  |  |  | (0.000) |  |  |  |  |  | (0.000) |
| X Middle |  |  |  |  | -0.0001* | X Woman |  |  |  |  | 0.0000 |
|  |  |  |  |  | (0.000) |  |  |  |  |  | (0.000) |
| X Old |  |  |  |  | -0.0002 |  |  |  |  |  |  |
|  |  |  |  |  | (0.000) |  |  |  |  |  |  |
| Constant | 0.1460*** | 0.1227*** | 0.1350*** | 0.0222 | 0.1376*** | Constant | 0.1461*** | 0.1083*** | 0.1260*** | 0.0052 | 0.1223*** |
|  | (0.030) | (0.032) | (0.030) | (0.046) | (0.030) |  | (0.025) | (0.026) | (0.026) | (0.049) | (0.025) |
|  |  |  |  |  |  |  |  |  |  |  |  |
| Number of observations | 1,305 | 1,305 | 1,302 | 1,302 | 1,302 | Number of observations | 870 | 870 | 868 | 868 | 868 |
| R-squared | 0.9050 | 0.9062 | 0.9066 | 0.9078 | 0.9090 | R-squared | 0.9119 | 0.9143 | 0.9130 | 0.9142 | 0.9165 |

Notes: See table 3.

**Table S3.** Macro-level determinants of the education-gap in unemployment vs. employment between low and high levels of education (including an interaction between EPL and GDP change)

|  | All |
| --- | --- |
| GDP change | -0.0063*** |
|  | (0.002) |
| GDP level | -0.0063*** |
|  | (0.001) |
| Temporary employment | -0.0039*** |
|  | (0.001) |
| Public sector | 0.0045** |
|  | (0.001) |
| EPL regular | 0.0138 |
|  | (0.010) |
| EPL regular * GDP change | 0.0014* |
|  | (0.001) |
| Constant | 0.1865** |
|  | (0.056) |
|  |  |
| Number of observations | 323 |
| R-squared | 0.9423 |

Notes: See table 4.

**Figure S1.** Average marginal effects (difference in likelihood of unemployment between low and high educated persons) from logistic regression models

*
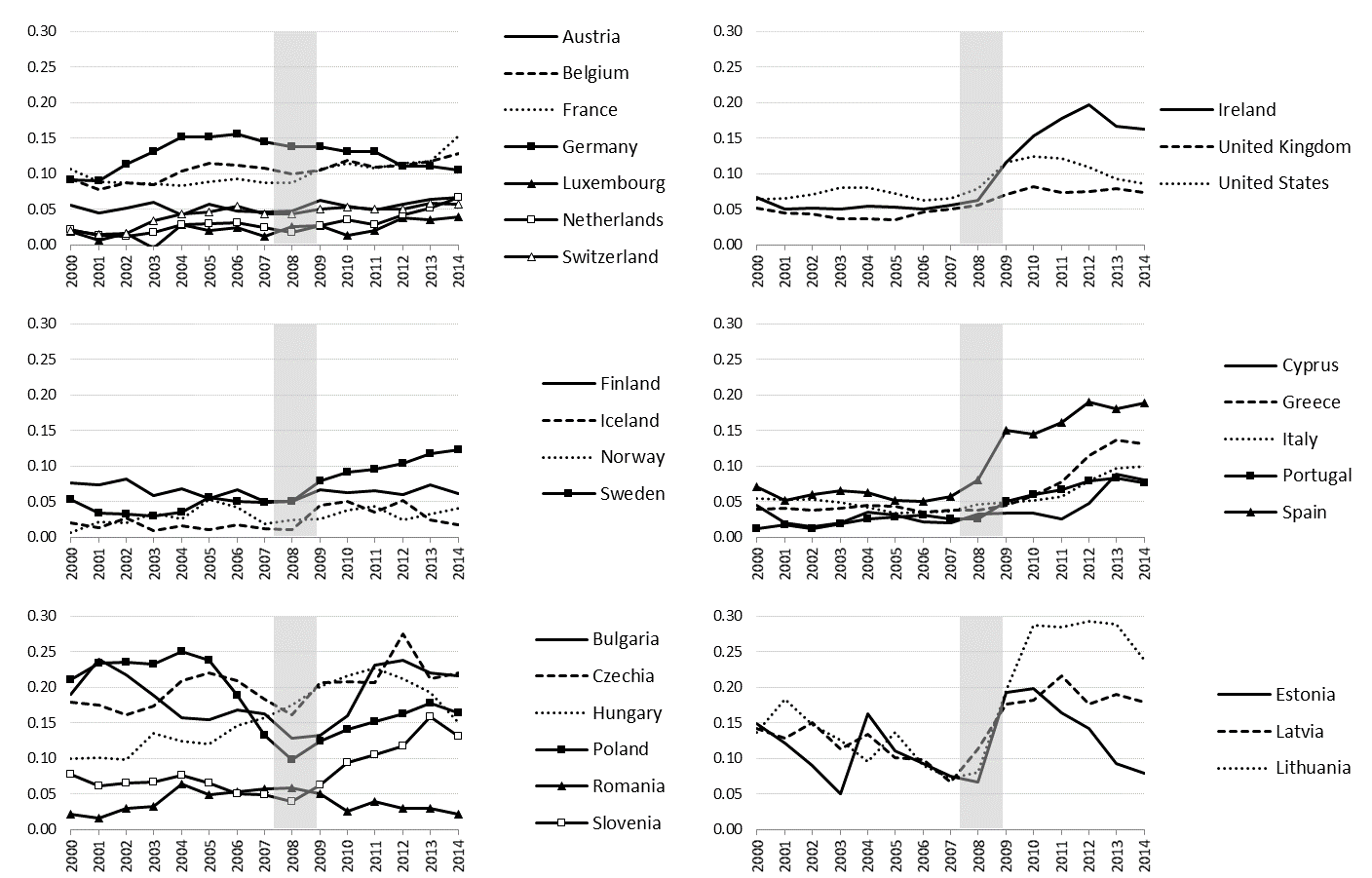
*

Notes: Grey shading indicates the year 2008, the beginning of the Great Recession. Slovakia is not shown because of very high numbers (AME 2000: 0.36; 2005: 0.48; 2010: 0.41; 2014: 0.38).

**Figure S2.** (A) Gap in the unemployment rate between low and medium educated persons (in percentage points) and (B) change in the unemployment rate gap between low and medium educated persons (in percentage points)

(A) (B)


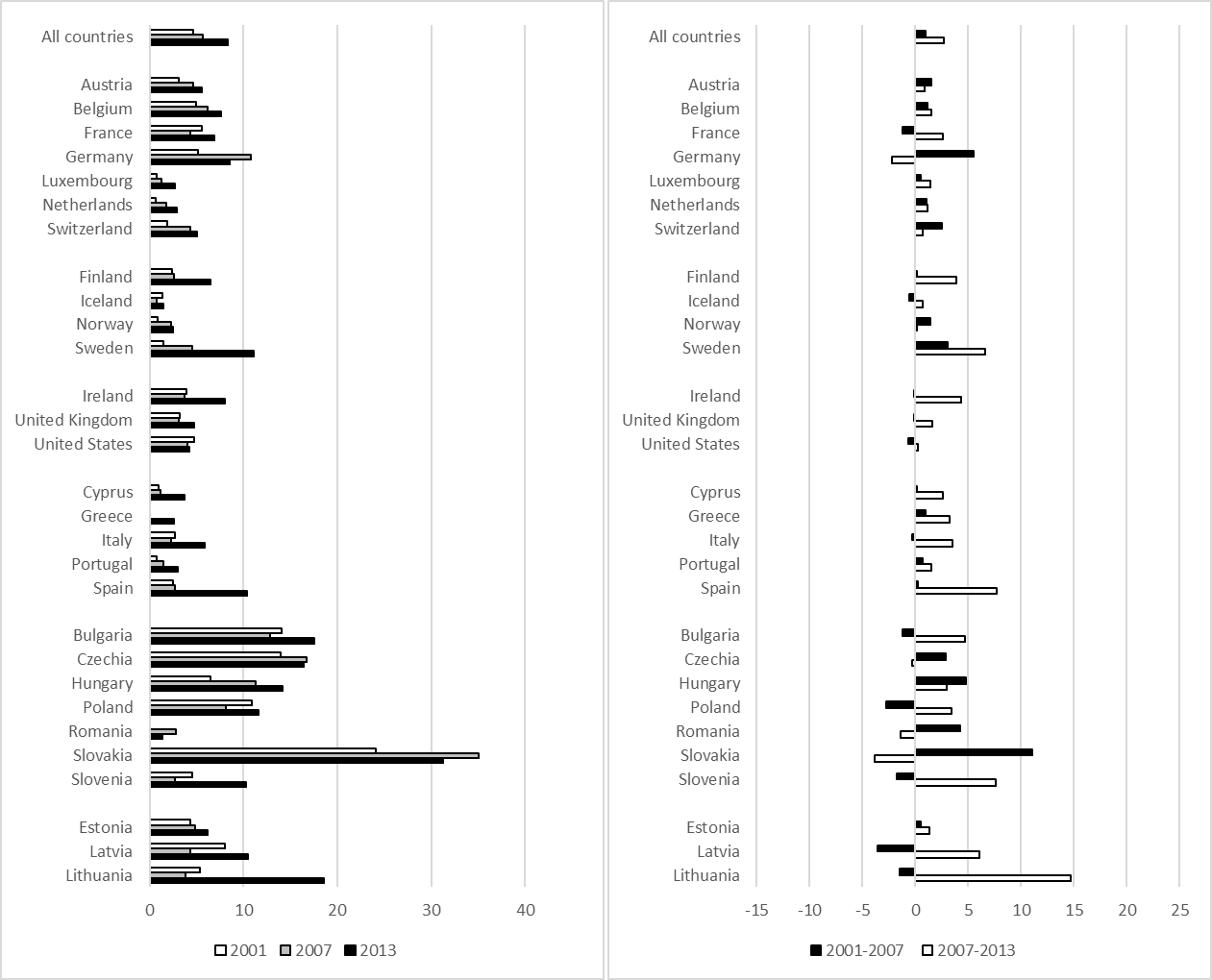

Supplement: sj-docx-1-asj-10.1177_00016993221083226 - Supplemental material for Growing inequality during the Great Recession: Labour market institutions and the education gap in unemployment across Europe and in the United States [file sj-docx-1-asj-10.1177_00016993221083226.docx]
